# Longitudinal changes of circumpapillary retinal nerve fiber layer thickness profile during childhood myopia progression

**DOI:** 10.1038/s41598-022-06489-w

**Published:** 2022-02-15

**Authors:** Ahnul Ha, Young Kook Kim, Sung Uk Baek, Jin-Soo Kim, Jin Wook Jeoung, Ki Ho Park

**Affiliations:** 1 Department of Ophthalmology, Seoul National University College of Medicine, Seoul, Korea; 2 Department of Ophthalmology, Jeju National University Hospital, Jeju-si, Korea; 3 Department of Ophthalmology, Jeju National University College of Medicine, Jeju-si, Korea; 4 Department of Ophthalmology, Seoul National University Hospital, Seoul, Korea; 5 Childhood Glaucoma Division, Department of Pediatric Ophthalmology, Seoul National University Children’s Hospital, Seoul National University College of Medicine, 101 Daehak-ro, Jongno-gu, Seoul, 03080 Korea; 6 Department of Ophthalmology, Hallym University College of Medicine, Chuncheon, Korea; 7 Department of Ophthalmology, Hallym University Sacred Heart Hospital, Anyang, Korea; 8 Department of Ophthalmology, Chungnam National University Sejong Hospital, Sejong, Korea

**Keywords:** Optic nerve diseases, Refractive errors, Paediatric research, Medical imaging

## Abstract

The purpose of this study was to evaluate longitudinal changes of circumpapillary retinal nerve fiber layer thickness (cpRNFLT) profile arising in the course of childhood myopia progression. Thirty-six eyes of 36 healthy children who showed myopia progression (spherical equivalent [SE] decrease of ≥ 2.0 diopters [D]) were included. To account for the axial-elongation-induced magnification effect on spectral-domain optical coherence tomography (SD-OCT) measurements, we calculated the proportion of quadrant-cpRNFLT distribution (i.e., the percentage of cpRNFLT within a single quadrant of total cpRNFLT). During 4.1 ± 1.1 years, the mean SE changed from -1.3 ± 0.9 to -4.3 ± 0.8D, and both the optic disc tilt ratio and the torsional angle increased (both *P* < 0.001). In the temporal quadrant, the cpRNFLT proportion was increased from 19.2 ± 1.86 to 24.4 ± 2.30% (*P* < 0.001). The cpRNFLT proportion in 3 quadrants (i.e., superior, inferior, nasal) showed decreases (all *P* < 0.001). Between baseline and follow up, the scan-circle location as determined by OCT was shifted mostly (94%; 34 of 36 eyes) toward the nasal side of the optic disc. With scan-circle repositioning to match the baseline, cpRNFLT distribution proportions did not show any significant difference between the baseline and follow up (all *P* > 0.05). For longitudinal evaluations of patients with myopia progression, scan-circle alteration should be given due consideration.

## Introduction

Myopia, a refractive error occurring primarily due to excessive axial elongation of the eye, is one of the most common eye conditions of pediatric populations worldwide^1^. The significant association between myopia and glaucoma is already well recognized^2–4^. The risk of developing glaucoma is as much as two to three times higher in myopias than in nonmyopias^3,5^. Since axial elongation in myopic eyes tends to be accompanied by structural optic nerve head (ONH) and peripapillary area alterations where glaucomatous damage is incurred^6–10^, the myopic optic disc provides information that can expand our understanding of glaucoma pathophysiology^11–13^.

The utility of spectral-domain optical coherence tomography (SD-OCT) for quantitative, rapid and non-invasive evaluation of circumpapillary retinal nerve fiber layer thickness (cpRNFLT) has established this imaging modality as an indispensable tool for diagnosis and monitoring of glaucoma progression^14,15^. The effects of myopia on cpRNFLT measurements by OCT have been studied^16–19^. Kang et al. showed that in young myopic eyes without additional ophthalmic abnormalities, axial length (AXL) affects average cpRNFLT and its distribution^20^. They also reported that with longer AXL, temporal cpRNFLT increased while the other sectors thicknesses decreased. Leung et al., having analyzed SD-OCT data on 189 healthy myopic participants, showed that the RNFL distribution angle on an RNFL thickness map decreased with increasing myopia^21^.

It should be noted, however, that those studies had cross-sectional designs, which limited the insights that could be drawn from their data regarding the time courses of cpRNFLT changes associated with myopia development and progression. To date, there are still no longitudinal reports on cpRNFLT changes during myopia progression or how such changes correlate with ONH-morphologic alterations.

Accordingly, in this study, we investigated SD-OCT-obtained longitudinal changes of cpRNFLT associated with myopia progression in children. Specifically, we analyzed data on cpRNFLT, distribution profiles, and ONH-morphologic alterations.

## Results

Initially, 39 eyes of the total of 39 patients who had met the eligibility criteria were included. Among them, 2 with poor-quality fundus photography (1 also had OCT artifact) and 1 who was diagnosed with associated uveitis over the course of the follow up were excluded from further analysis. Finally, 36 eyes of 36 children (20 males, 16 females) were included in the final analysis. Ten (10) of them had increased CDR in one or both eyes, and 11 had a family history of glaucoma. The remaining 15 were cases for routine ophthalmic examination due to myopia.

### Demographic and clinical characteristics

The study population’s demographics and clinical characteristics are provided in online supplementary eTable S1. At baseline, the mean age was 8.9 ± 2.1 years. The mean SE of the refractive errors was − 1.3 ± 0.9 D (range: − 3.5 to 0.0). The baseline cup-to-disc ratio (CDR) was 0.5 ± 0.1 (range: 0.2–0.7), and no cases of cupless disc were included.

After an average 4.1 ± 1.1-year follow up, the mean SE became − 4.3 ± 0.8 D (range: − 6.1 to − 3.0). Compared with the baseline, the mean SE was significantly decreased, and both the optic disc tilt ratio and the torsional angle (in absolute values) were significantly increased (all *P* < 0.001). The inter-observer ICCs for the optic disc tilt ratio and torsional angle measurements were 0.844 (95% CIs 0.743–0.905, *P* < 0.001) and 0.977 (95% CIs 0.961–0.986, *P* < 0.001), which indicated good-to-excellent inter-observer reproducibilities^22^.

### Longitudinal changes of cpRNFLT and topographic profile

Online supplementary eTable S2 compares the baseline and follow-up values of the SD-OCT cpRNFLT measurements. At the final visit, the average cpRNFLT was 96.8 ± 2.59 µm, which represented a decrease relative to the baseline. The cpRNFLT in 3 of the quadrants (i.e., superior, inferior, and nasal) showed statistically significant decreases. The thickness changes in each quadrant were − 8.80 ± 7.89 µm (*P* < 0.001) in the superior, − 6.31 ± 8.56 µm (*P* < 0.001) in the inferior, and − 7.67 ± 7.93 µm (*P* < 0.001) in the nasal, respectively. Meanwhile, the cpRNFLT in the temporal quadrant was 94.7 ± 9.44 µm, which was a significant increase (change: + 19.3 ± 6.01 µm) relative to the baseline (*P* < 0.001).

The longitudinal changes of the quadrant-cpRNFLT distribution are provided in online supplementary eTable S3. The cpRNFLT proportion in the superior, inferior, and nasal quadrants was lower according to the SD-OCT obtained at the final visit. The differences between the baseline and follow up were − 1.99 ± 1.85% (range: − 7.42 to 0.22; *P* < 0.001) in the superior, − 1.28 ± 2.02% (range: − 4.76 to 3.23; *P* = 0.001) in the inferior, and − 1.90 ± 2.12% (range: − 7.19 to 1.12; *P* < 0.001) in the nasal quadrant. In the temporal quadrant, however, the cpRNFLT proportion was significantly increased, from 19.2 ± 1.86% (range: 16.5–23.8) to 24.4 ± 2.30% (range: 20.4–30.2; *P* < 0.001).

### Changes of SD-OCT scan-circle location

**Figure 1:**
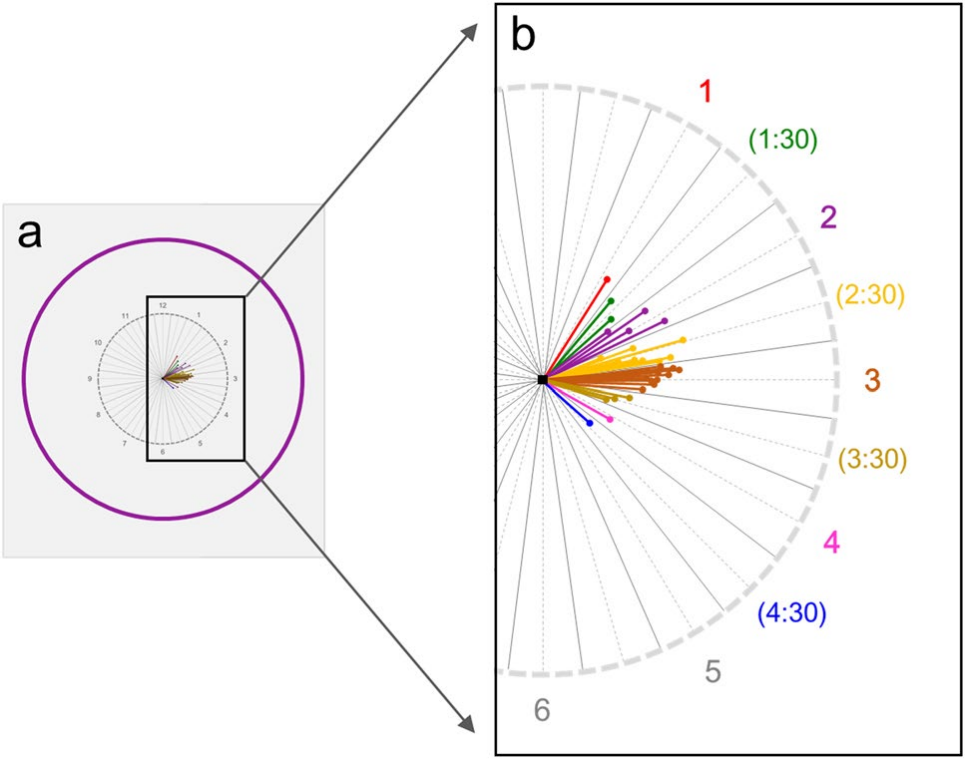
Diagram showing directional vectors of scan-circle center movement from baseline to final visit in study subjects. (**a**) Direction and amount of center shift presented in SD-OCT scan circle (purple solid circle: SD-OCT scan circle; purple dotted circle: virtual optic disc margin). The distance of center shift was calculated as the relative length compared with the scan-circle diameter. Also, the disc-fovea axis was aligned in the 3 o’clock direction in each case. (**b**) Magnification image of black square in (**a**). The major directionality of the scan-circle shift was to the nasal side of the optic disc. The scan-circle center movement was most frequent toward 3 o’clock (n = 14), followed by 2.5 (n = 10) and 2 o’clock (n = 4).

Figure 1 shows directional vectors of scan-circle center movement from the baseline to the final visit in study subjects. The major directionality of the scan-circle shift was to the nasal side of the optic disc. The scan-circle center movement was most frequent toward 3 o’clock (n = 14), followed by 2.5 (n = 10) and 2 o’clock (n = 4).

### cpRNFLT and distribution after scan-circle repositioning

Table 1 provides the cpRNFLT and distribution proportions after adjustment of scan-circle location on final SD-OCT. The average as well as quadrant cpRNFLT significantly differed between the baseline and the final visit with scan-circle repositioning.

**Table 1:** Circumpapillary retinal nerve fiber layer thickness and distribution proportions after adjustment of scan-circle location on final optical coherence tomography.

	First visit (*A*)	Last visit (*B*)	Last visit (repositioning; *C*)	*P* value*	Contrasts TEST
**Thickness (µm)**					
Global	97.7 ± 3.26 (93–104)	96.8 ± 2.59 (92–104)	97.0 ± 3.31 (92–104)	0.026	0.021 (A > B), < 0.001 (A > C), 0.999 (B = C)
**Quadrants**					
Superior	121.6 ± 6.28 (108–131)	112.8 ± 7.04 (100–123)	120.6 ± 5.72 (108–128)	< 0.001	< 0.001 (A > B), 0.034 (A > C), < 0.001 (B < C)
Temporal	75.4 ± 7.65 (62–98)	94.7 ± 9.44 (80–118)	75.4 ± 7.83 (62–100)	< 0.001	< 0.001 (A < B), 0.026 (A > C), < 0.001 (B > C)
Inferior	128.04 ± 5.72 (113–139)	122.1 ± 8.48 (104–139)	126.8 ± 5.95 (112–128)	< 0.001	< 0.001 (A > B), < 0.001 (A > C), 0.015 (B < C)
Nasal	65.2 ± 8.61 (45–79)	57.5 ± 8.38 (40–73)	65.2 ± 7.88 (48–78)	< 0.001	< 0.001 (A > B), 0.018 (A > C), < 0.001 (B < C)
**Distribution proportion (%)**					
Superior	31.2 ± 1.29 (27.8–33.5)	29.3 ± 1.77 (24.8–31.7)	31.1 ± 1.22 (27.9–33.3)	< 0.001	< 0.001 (A > B), 0.288 (A = C), < 0.001 (B < C)
Temporal	19.2 ± 1.86 (16.5–23.8)	24.4 ± 2.30 (20.4–30.2)	19.5 ± 1.78 (16.5–24.6)	< 0.001	< 0.001 (A < B), 0.158 (A = C), < 0.001 (B > C)
Inferior	32.8 ± 1.16 (30.0–36.1)	31.6 ± 2.00 (27.4–35.5)	32.7 ± 1.16 (30.2–35.5)	0.002	0.003 (A > B), 0.335 (A = C), 0.010 (B < C)
Nasal	16.7 ± 2.04 (11.7–20.8)	14.8 ± 2.07 (10.8–19.5)	16.5 ± 1.91 (12.4–20.2)	< 0.001	< 0.001 (A > B), 0.990 (A = C), < 0.001 (B < C)

Values are mean ± standard deviation (range).

Bonferroni correction for multiple comparisons was applied.

*Repeated measures ANOVA with Greenhouse–Geisser correction.

**Figure 2:**
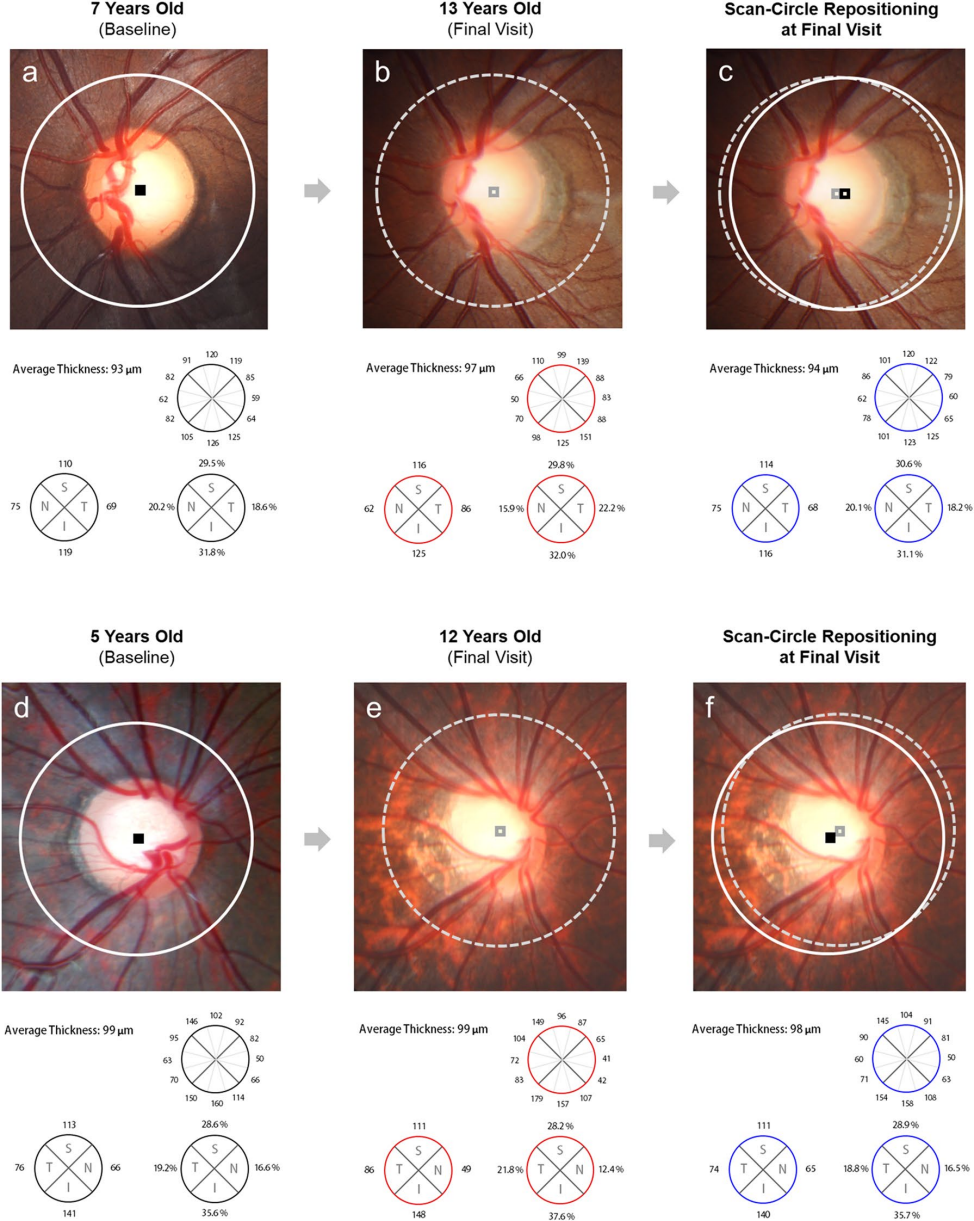
Representative cases showing change of circumpapillary retinal nerve fiber layer thickness (cpRNFLT) distribution during childhood myopia progression. (**a**–**c**) During the 6-year follow up (from 7 to 13 years old), SE decreased from − 0.75 D to − 4.00 D. The optic-disc tilt ratio increased by 13.5% (from 1.11 to 1.26). (**a**) Relative to the baseline examination, (**b**) the cpRNFLT profile changed with increasing temporal cpRNFLT (from 18.6 to 22.2%) and the decrease of the other 3 quadrants according to progressive optic-disc tilting. (**c**) After scan-circle repositioning, the cpRNFLT profile of the follow-up scan was similar to that of the baseline examination. (**d**–**f**) During the 7 years’ follow up (from 5 to 12 years old), SE decreased from − 0.5D to − 5.0 D. In this case, not only did optic-disc tilt ratio increase (from 1.01 to 1.30), but torsional angle did as well (inferiorly, from 1.60° to 14.5°). As was the case with progressive optic-disc tilt and torsion, the cpRNFLT profile increased in the temporal quadrant (**e** from 19.2 to 22.1%) relative to the baseline examination (**d**). (**f**) After scan-circle repositioning to match the baseline’s scan-circle center, the cpRNFLT profile showed a more-similar pattern to that of the baseline OCT.

However, the distribution proportions of quadrant cpRNFLT did not show significant differences between the baseline and the final visit with scan-circle repositioning in any of the 4 quadrants (superior: *P* = 0.288; temporal: *P* = 0.158; inferior: *P* = 0.335; nasal: *P* = 0.990). The inter- and intra-examiner reproducibilities of scan-circle positioning showed good-to-excellent agreement (online supplementary eTables S4 and S5). Figure 2 shows representative cases of cpRNFLT distribution during childhood myopic progression.

### Factors associated with changes of cpRNFLT distribution

The univariate analysis revealed that greater SE change, greater tilt-ratio increase, and greater torsional angle increase were associated with greater change of cpRNFLT distribution without scan-circle repositioning. In multivariate analysis, greater change of tilt ratio was associated significantly with greater cpRNFLT distribution change (*P* = 0.047). The full summary of statistical results is available in Table 2.

**Table 2:** Univariate and multivariate mixed-effects model determination of factors associated with changes of circumpapillary retinal nerve fiber layer thickness (cpRNFLT) distribution during childhood myopic progression.

Variables	Univariate analysis				Multivariate analysis			
	Estimate	Standard error	95% confidence interval	*P*	Estimate	Standard error	95% confidence interval	*P*
Baseline age (years)	− 0.210	0.229	− 0.678 to 0.258	0.368				
Total follow-up period (years)	0.015	0.490	− 0.987 to 1.016	0.976				
SE at the baseline (D)	0.479	0.522	− 0.586 to 1.544	0.366				
Δ SE (D)	1.140	0.647	− 0.183 to 2.462	0.089	0.075	0.993	− 1.959 to 2.108	0.941
Δ Tilt ratio (%)	0.158	0.076	0.003 to 0.313	0.048	0.158	0.076	0.035 to 0.208	0.047
Δ Torsional angle (°)	0.291	0.163	− 0.043 to 0.624	0.085	0.177	0.179	− 0.189 to 0.988	0.331

*SE* spherical equivalent, Δ change.

The results show factors associated with greater change in cpRNFLT distribution without scan-circle repositioning.

The ‘change of cpRNFLT distribution’ was defined as the sum of differences in distribution proportion in all four quadrants between the baseline and the final visit (in absolute values).

This study obtained and evaluated longitudinal Cirrus SD-OCT data on cpRNFLT changes occurring during myopia progression in children. The principal findings are as follows. First, in the course of myopia progression, the cpRNFLT distribution in 3 of the quadrants (i.e., superior, inferior, nasal) was decreased, whereas that in the temporal quadrant was significantly increased. Second, the scan-circle location determined by the SD-OCT built-in algorithm was significantly changed. Third, following scan-circle repositioning to match the baseline location, the cpRNFLT distribution was not statistically different from the baseline’s.

The ONH has long been implicated as a critical site in the initiation of glaucoma^23^. With regard to myopic eyes, it has been suggested that altered biomechanical properties of structures around the ONH accompanied by axial elongation might be related to the pathogenesis of glaucomatous damage^24^. Such myopic-ONH-associated morphological alterations include optic disc tilt, torsion, focal lamina cribrosa defect or tilt, and parapapillary atrophy (PPA). Also, differing ONH anatomy makes accurate diagnosis of glaucoma in myopic eyes more challenging.

Previous cross-sectional studies conducted with adults have reported that myopic eyes can have a cpRNFLT topographic profile differing from that of non-myopic eyes^20,25–27^. Kim et al., having measured 48 myopic eyes by Stratus OCT (Carl Zeiss Meditec), demonstrated that the temporal cpRNFLT was thicker in a moderate-and-high myopia group (SE ≤ − 3.0 D) than in a low-myopia group (3.0 D < SE < 0.0 D)^28^. Also, Kang et al. measured cpRNFLT in 269 young subjects (age under 26 years) by Cirrus SD-OCT, and noted a positive correlation between AXL and temporal cpRNFLT^20^. Hong et al., likewise, demonstrated that cpRNFLT peaks were closer at the temporal quadrant with increasing myopia^29^. As in previous reports, our longitudinal results showed that the temporal-quadrant cpRNFLT distribution (without scan-circle repositioning) significantly increased during childhood myopia progression.

The reason for such a different cpRNFLT topographic profile in myopic eyes has remained unclear. It has been hypothesized that retinal dragging toward the temporal horizon, accompanied by axial elongation, might be among the underlying factors^28^. These changes can be detected as a straightening and a shift toward the fovea of the RNFL bundles as well as the retinal vessels on fundus photography^30^. Another proposed explanation is that the superior and inferior RNFL bundle position moves closer to the macula as the result of asymmetrical anteroposterior elongation or posterior protrusion in myopic eyes^21^. Previous studies imaging the shape of the globe in eyes with high myopia using magnetic resonance imaging have shown that with myopia increase, eye dimensions increased in all directions such that the increase in length was considerably greater than the increases in width and height^31,32^. Furthermore, emmetropic retinas’ oblateness also decreased with myopia increase^32^. It is plausible that the distribution of the RNFL bundles conforms to the shape of the globe. Longitudinal changes in globe shape and accompanied alterations in retinal shape might affect the peak RNFL position. Leung et al. also suggested that temporal convergence of RNFL bundles could be an image artifact that is implicated in increased vertical curvature of the retina^21^.

This study suggests that another probable reason for myopic eyes’ markedly different cpRNFLT topographic profile is a change of OCT scan-circle location according to morphologic alteration of the ONH during myopia progression. In our child subjects with myopia progression, the cpRNFLT distribution profile showed very close agreement with the baseline following scan-circle repositioning. In eyes with tilted disc, the temporal margin determined by the Cirrus OCT’s built-in algorithm was significantly shifted nasally, thus resulting in an overall nasally deviated scan-circle center position. Nasal scan-circle displacement increases temporal-region thickness and decreases non-temporal-region thickness^33^. This fact also corresponds well with a previous finding that temporalization of SD-OCT scan circle in adults can significantly reduce false-positive detection of myopic tilted discs^34^.

Given the significant association between myopia and glaucoma, cpRNFLT distribution pattern difference in myopic eyes on SD-OCT already has important implications for RNFL thickness map interpretation. It is also critical to determine, in terms of both myopia progression and glaucoma deterioration on SD-OCT, structural changes that occur during axial elongation. Although we showed that the scan circle had been nasally shifted in eyes with myopia relative to the baseline, this fact does not necessarily mean that scan-circle position should be corrected in all myopic adults when evaluating cpRNFLT. This is due to the fact that most patients might not have had OCT scans taken at an early age prior to the onset of myopia, and thus, for them, determination of the baseline scan-circle position would be impossible. Although morphological alterations of the ONH over time tend to be unremarkable in most adults^35^, there clearly are adult patients with myopia showing longitudinal changes in the ONH. In these cases, comparing cpRNFLT changes according to the scan-circle position determined on the first OCT would be one of the important factors enabling obtainment of reliable results. Moreover, in children, especially those with significant axial elongation and accompanying ONH-morphologic changes, scan-circle location as determined by OCT’s built-in algorithm should be evaluated carefully for measurement reproducibility in follow-up examinations.

In the current study, the torsional angle significantly increased during the 4-year period. Since we included children who showed myopia progression, most of the participants showed morphologic ONH alterations accompanied by axial elongation. In a longitudinal study including 173 Korean children, 42 (24.3%) showed progressive optic disc torsion greater than 5 degrees during the mean follow-up period of 3.7 years. In the same study, development of optic disc torsion was associated with greater myopic shift, optic disc tilt, and increased PPA width^36^. In our results, cpRNFLT in the superior and inferior quadrants was less affected by scan-circle-position change than was that in the temporal and nasal quadrants. However, we believe that the patterns of morphologic change in the ONH during myopia progression might be an important factor determining those of cpRNFLT change. Most of the children included in our study showed nasal shifting of the scan-circle position (± 15°, 94%). Change in optic disc torsional angle between the baseline and the last follow up was about 7 degrees on average. In cases with greater alterations in torsional angle, the position of the scan circle might move in the oblique direction, causing greater changes in superior and inferior cpRNFLT.

Although our current study provides the first detailed longitudinal analysis of cpRNFLT distribution change in the course of childhood myopia progression, several limitations need to be considered. First, it should be noted that the magnification effect was not adjusted according to AXL, since some subjects did not have longitudinal AXL data. The thinner cpRNFLT in the follow-up (both with and without scan-circle repositioning) might have resulted from ocular magnification to some extent. Even if we had adjusted for magnification, the effect of normal childhood development on cpRNFLT should also have been taken into account. However, there have not been sufficient studies investigating the course of physiologic cpRNFLT changes throughout childhood. Thus, we were unable to compare, directly, cpRNFLT as measured at different time points. Therefore, we focused on analyzing cpRNFLT distribution *proportions* rather than cpRNFLT itself in each individual. We assumed that although the overall cpRNFLT was affected by axial elongation or the magnification effect, its proportion to the total thickness would be less affected unless the distribution of RNFL were truly changed. This notwithstanding, future studies considering magnification and aging effects on cpRNFLT during myopia progression are essential. Also, we could not evaluate any detailed effects of other parameters such as CDR, PPA, or corneal astigmatism on scan-circle center movements. Notably, tilted discs are associated with both myopia and astigmatic refractive errors^37,38^. Second, this study included children with a large CDR (between 0.6 and 0.7; n = 10 eyes; 28%) or family history of glaucoma. This factor needs to be considered when interpreting our results, notwithstanding the fact that those individuals did not manifest any signs of glaucoma during the average 4.1-year follow up. Third, its retrospective design has inherent possible biases. Finally, additional limitations of the present study are that its number of participants was limited, it did not include subjects in early childhood, and its follow-up period did not extend until adulthood. Studies of small sample size tend to yield lower statistical power for detection of true associations^39^. It is possible that greater axial elongation results in further cpRNFLT distribution changes, which could not be observed in this study. Definitely, future prospective studies with large numbers of participants are needed.

In summary, this study demonstrated that temporal-quadrant cpRNFLT distribution, as determined by Cirrus SD-OCT, significantly increased during childhood myopia progression. However, the cpRNFLT distribution changes were due mainly to the nasal shift of the scan-circle position in myopic tilted disc. False-positive changes on OCT-obtained cpRNFLT should be given due consideration in longitudinal evaluations of patients presenting with myopia progression.

## Methods

This study was approved by the Institutional Review Board of Seoul National University Hospital, and fully adhered to the Declaration of Helsinki. Informed consent was waived by the ethics committee due to the study’s retrospective nature.

### Study cohort

For establishment of the study’s cohort, Clinical Data Warehouse of Seoul National University Hospital Patients Research Environment (SUPREME) was used. It included electronic medical records on children who had visited the Seoul National University Children’s Hospital (compiled between January 2012 and January 2020), which were retrospectively reviewed.

The specific inclusion criteria were as follows: (1) 13 years or younger at baseline examination; (2) at least 2 sufficient-quality SD-OCT images that had been taken at intervals of more than 3 years; (3) myopic progression defined as decreased cycloplegic spherical equivalent (SE) refraction ≥ 2.0 diopters (D) during the 2 SD-OCT examination intervals.

Participants were examined for best-corrected visual acuity assessment, cycloplegic refraction, intraocular pressure (IOP) measurement by non-contact tonometry, color fundus photography, AXL measurement (Axis II PR; Quantel Medical, Inc., Bozeman, MT, USA) and Cirrus high-definition SD-OCT (version 6.0; Carl Zeiss Meditec, Dublin, CA, USA). Cycloplegic refraction was performed after administration of Tropicamide 1% eye drops in each eye, one drop each after 10 min (twice) to produce the maximum cycloplegic effect. One additional drop was instilled after 30 min if pupillary light reflex was present.

Patients were excluded for any of the following reasons: history of retinal disease; optic nerve disease (including glaucoma); history of ocular hypertension; history of systemic or neurological diseases. The presence of glaucoma was judged according to the baseline as well as examination records representative of the entire follow-up. The glaucoma diagnosis had been made based on the following criteria^40^: (1) characteristic ONH change (progressive increased in CDR, CDR asymmetry ≥ 0.2, and focal neuroretinal rim thinning); (2) intraocular pressure > 21 mmHg; (3) presence of corresponding glaucomatous visual field defects, which was defined as (1) a cluster of ≥ 3 points with *P* < 0.05 on a pattern deviation map in at least one hemifield, including ≥ 1 point with *P* < 0.01, (2) a pattern standard deviation of *P* < 0.05, (3) or a glaucoma hemifield test result outside the normal limits, as confirmed by at least 2 examinations. Subjects meeting 2 or more of the above-noted criteria were diagnosed with glaucoma and thus, were excluded from our study. Those with only increased CDR or a family history of glaucoma were not excluded.

### OCT image acquisition

All of the images were obtained through dilated pupils by the same experienced examiner. A 200 × 200 optic-disc cube scan protocol was applied for measurement of the cpRNFLT within a 6 × 6 mm^2^ area in the region of the optic disc. A built-in algorithm located the center of the optic disc, even in cases where it was not well-centered^41^. The disc center was identified according to the determination of a dark spot near the scan center, which was of a shape and size consistent with the normal range for optic discs^21^. Then, a 3.46 mm diameter calculation circle consisting of 256 A-scans was positioned around the optic disc, after which the average cpRNFLT was determined. All of the scans were reviewed by an experienced ophthalmologist (YKK) for segmentation errors and image artifacts. Only high-quality scans (signal strength ≥ 7, absence of discontinuity or misalignment, no involuntary saccade, blinking artifacts, segmentation failure or artifacts) were applied to the final analysis.

### Proportion of quadrant-cpRNFLT distribution

In this study, we focused on longitudinal changes of the *proportion* of the cpRNFLT distribution profile as well as cpRNFLT itself, in each individual. For that purpose, we calculated the proportion of the quadrant-cpRNFLT distribution using the formula below. All of the data for the left eye were flipped to the right-eye orientation to permit comparison between quadrants from the left and right eyes.

$${\text{Proportion of Quadrant cpRNFLT }} = \, \left( {\text{Superior, Nasal, Inferior or Temporal}} \right){\text{ cpRNFLT }}/{\text{ total cpRNFLT}} \times 100 $$

### Measurement of ONH parameters

The fundus photographs were assessed independently by 2 glaucoma specialists (AH/JSK) in a masked fashion without knowledge of any clinical information. Only photographs with adequate quality and clarity were included. The vertical CDR was measured using ImageJ software (V.1.48; Wayne Rasband, National Institutes of Health, Bethesda, Maryland, USA). Measurements of vertical disc diameter excluded areas of PPA and the Elschnig scleral ring. The vertical diameter of the cup was measured as the vertical distance between the points of maximal centrifugal extension of the cup between 11 to 1 o’clock and 5 to 7 o’clock^42^.

Optic-disc tilt ratio was measured on color fundus photographs as well as on the OCT-detected optic disc margin on the RNFL deviation map (supplementary eFigure S1). Torsional angle was measured on color fundus photographs using ImageJ software (V.1.48). Optic-disc tilt ratio was measured as the ratio between the longest and shortest diameters of the optic disc^43^. Optic-disc torsional angle was defined as that between the vertical meridian and the optic disc’s long axis. The vertical meridian was identified as a vertical line 90 degrees from the horizontal line connecting the fovea to the center of the optic disc (supplementary eFigure S1)^44^. In one case with the optic disc having exactly the same length in the long- and short-axis, we considered the vertical axis of the image to correspond to the optic disc’s long axis. For the final analysis, the average value of 2 independent measurements was used. In cases where the average difference between the 2 measurements differed by more than 15%, a third examiner (YKK) confirmed the measurement.

### Measurement of changes of SD-OCT scan-circle location

On closer examination of longitudinal SD-OCT images, we found that the scan-circle center location determined by SD-OCT’s algorithm significantly differed between the baseline and follow-up examinations. Morphologic alterations of the ONH during myopia progression can affect determination of the optic-disc center, with the consequence that the scan-circle position, which is automatically set 3.46 mm from the optic-disc center, might be changed as well. We therefore undertook to quantify the degree of optic-disc center location change between the baseline and final follow-up SD-OCT images.

**Figure 3:**
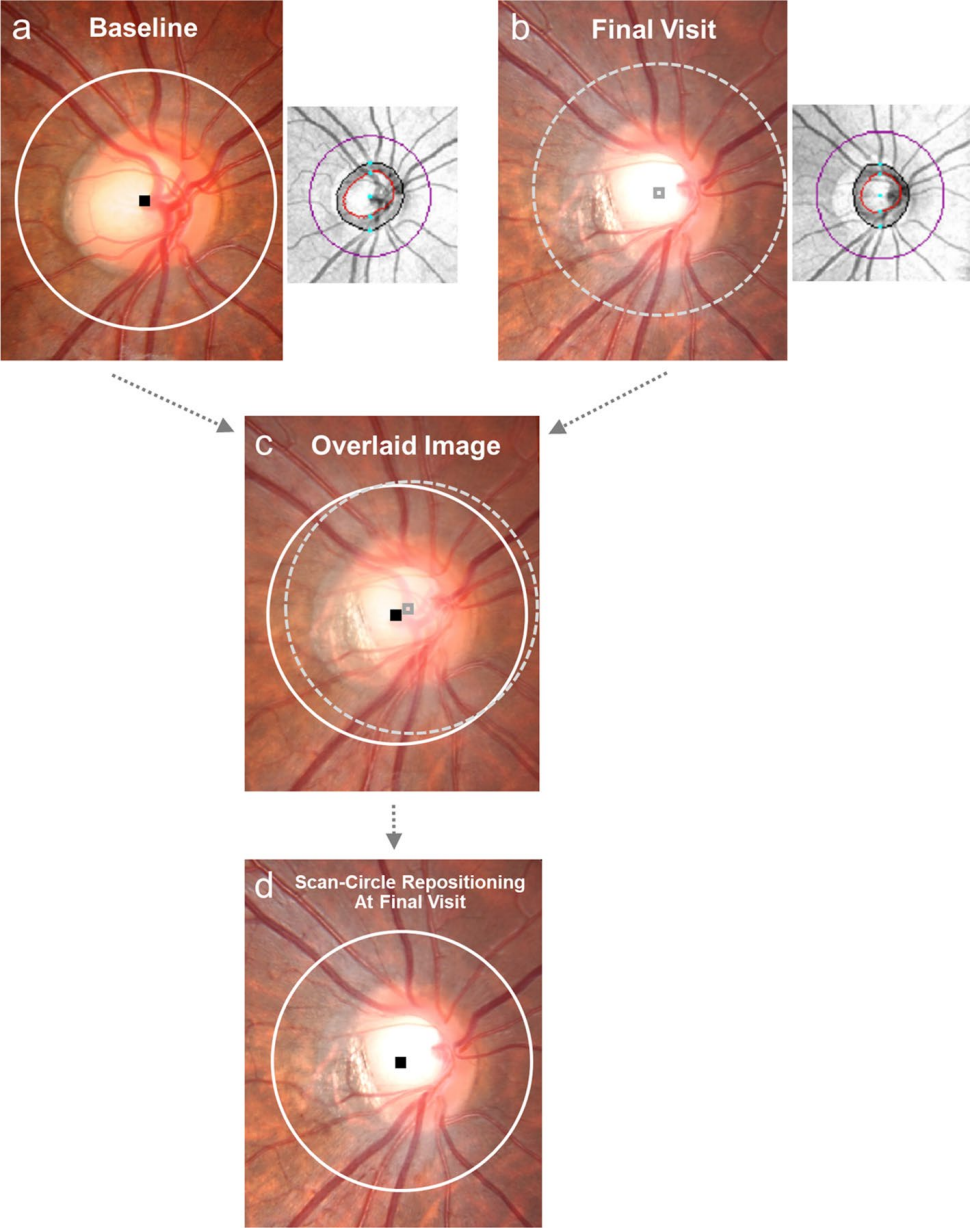
Determination of scan-circle repositioning location. (**a**) The center of the scan circle determined by spectral-domain optical coherence tomography (SD-OCT)’s built-in algorithm was displayed on the optic-disc photography at the time of the baseline examination. (**b**) The scan-circle center was shown also on optic-disc photography taken at the final visit. (**c**) The final disc photograph was overlaid onto the baseline disc photograph using the blood vessel contour around the optic nerve head as a reference. (**d**) Finally, by applying the optic-disc center (as determined on the baseline images) to the final disc photographs, a new scan-circle location was determined.

For that purpose, optic-disc-photograph overlay onto SD-OCT’s RNFL deviation map, as aligned by Photoshop software (Version 9.0; Adobe, San Jose, CA) based on vascular landmarks, was employed for determination of the optic-disc center at the baseline examination. Then, final disc photography was overlaid onto baseline disc photography, the blood vessel contour around the ONH being the reference (Fig. 3). By applying the optic-disc center determined on baseline images to the final disc photography, a new scan-circle location was determined. The direction of optic-disc center shift was recorded as clock-hour locations (all in the right-eye orientation). This process was conducted by a single experienced ophthalmologist (YKK).

Finally, the scan circle was newly positioned to match the baseline at the time of the final Cirrus SD-OCT examination. The Cirrus SD-OCT allows the controlled shifting of the 3.46-mm calculation circle with respect to the center of the ONH and does not require the user to obtain multiple scans^33^. For accurate positioning, first, we calculated the distance of scan-circle center movement in the overlaid fundus photographs by comparing the relative length with the scan-circle diameter. Then, according to the distance and direction of the vectors representing scan-circle center movement, the new location was determined. Finally, the position was re-confirmed based on the points where the scan circle meets the major blood vessels. This final procedure was performed independently by 2 glaucoma specialists (AH/SWB) in a masked fashion without knowledge of any other clinical information. For the final analysis, the cpRNFLT average value or distribution, as determined by 2 independent measurements, was used.

### Statistical analysis

The intra- and inter-examiner reproducibilities were evaluated by reference to the calculated intra-class correlation coefficients (ICCs) with their confidence intervals (CIs). Parametric or nonparametric tests were used, based on the normality of the data as assessed by Kolmogorov–Smirnov testing. Longitudinal cpRNFLT changes were evaluated using repeated measures ANOVA with analysis of margins and contrast. The harmonity of covariance was measured by Mauchly's test of sphericity. For cases of non-harmonized covariance state, the Greenhouse–Geisser test was used. We investigated how the cpRNFLT changes has been influenced by the demographic (baseline age, total follow-up period) and ocular factors (SE, tilt ratio, torsional angle), first by means of a univariate model and then by a multivariate model that included univariate-model variables for which *P* < 0.20. The analysis was performed using the SPSS statistical package (SPSS Advanced Statistics 21.0; Chicago, IL, USA). A 2-sided *P*-value < 0.05 was considered to signify statistical significance. In cases of multiple comparisons, the difference was evaluated after Bonferroni correction by multiplying *P* values by the number of associating factors.

## Supplementary Information

Supplementary Information.

## Data Availability

The dataset generated during the current study is available in the Supplementary Material.
