# Supplementary material for: Longitudinal changes of circumpapillary retinal nerve fiber layer thickness profile during childhood myopia progression
Source: Sci Rep. 2022 Feb 15;12:2555. doi: 10.1038/s41598-022-06489-w (PMC8847345; doi:10.1038/s41598-022-06489-w)
Supplement: Supplementary file 1 — Supplementary Information. [file 41598_2022_6489_MOESM1_ESM.docx]

**Supplementary Information**

**Longitudinal Changes of Circumpapillary Retinal Nerve Fiber Layer Thickness Profile during Childhood Myopia Progression**

Ahnul Ha, M.D, Young Kook Kim, M.D, Sung Uk Baek, M.D, Jin-Soo Kim, M.D, Jin Wook Jeoung, M.D, PhD, Ki Ho Park, M.D, PhD

**Online-Only Figure**

eFigure1. Measurement of optic disc tilt ratio and torsional angle

**Online-Only Table**

eTable 1. Demographic and Clinical Characteristics of Study Participants at Study Entry and Completion

eTable 2. Circumpapillary Retinal Nerve Fiber Layer Thickness Measures at Study Entry and Completion

eTable 3. Proportion of Quadrant Circumpapillary Retinal Nerve Fiber Layer Thickness Distribution at Study Entry and Completion

eTable 4. Inter-examiner Reproducibility of SD-OCT Scan-circle Repositioning

eTable 5**.** Intra-examiner Reproducibility of SD-OCT Scan-circle Repositioning

**Online-Only Figure**

**eFigure1. Measurement of optic disc tilt ratio and torsional angle.** Optic disc tilt ratio was defined as the ratio between the longest diameter (LD) and shortest diameter (SD) of the optic disc. Tilt ratio measured on (A) color fundus photography, and (B) OCT-detected optic disc margin in circumpapillary retinal nerve fiber layer deviation map. Optic-disc torsional angle (Ɵ) was defined as that between the vertical meridian (VM) and the optic disc’s long axis. The vertical meridian was identified as a vertical line 90 degrees from the horizontal line connecting the fovea to the center of the optic disc.

**
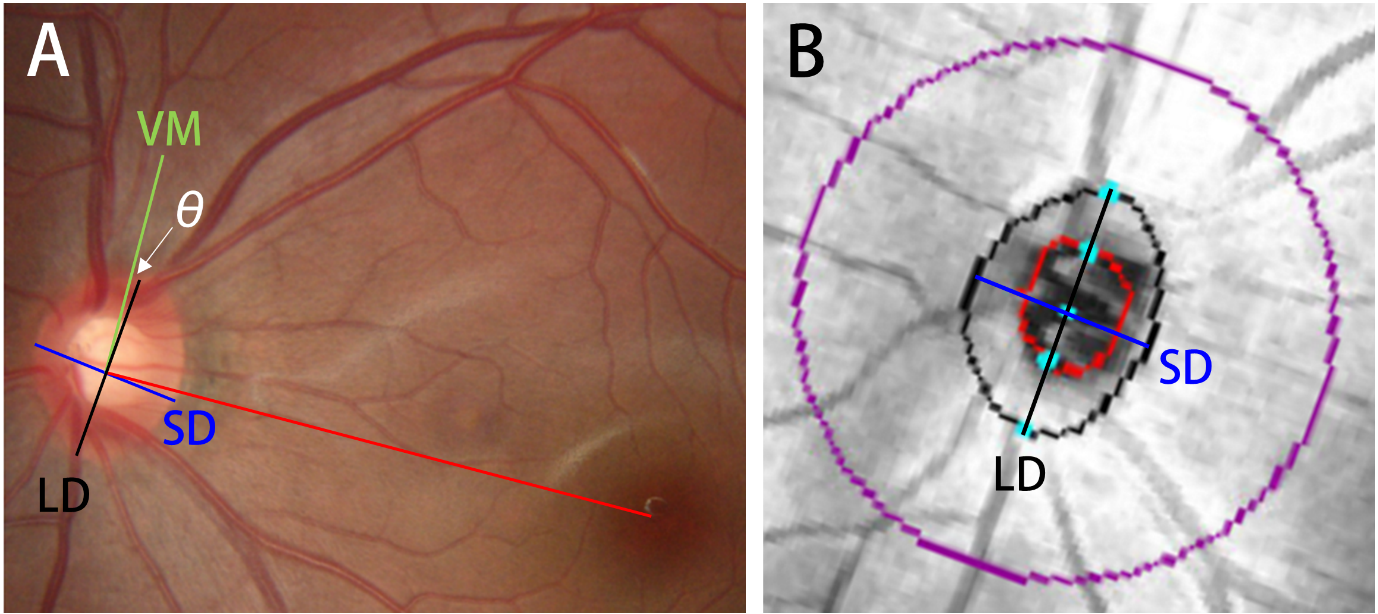
**

**Online-Only Table**

| **eTable 1.** Demographic and Clinical Characteristics of Study Participants at Study Entry and Completion | | | | |
| --- | --- | --- | --- | --- |
| **Characteristics** | **Study subjects** (*n* = 36) | | | |
|  | Baseline  (*First visit*) | Follow up  *(Last visit*) | Change *(Last visit - First visit)* | *P* value |
| Age, yrs | 8.9 ± 2.1 (5.7-12.5) | 13.0 ± 2.0 (10.2-15.9) |  | |
| Follow-up duration, yrs | 4.1 ± 1.1 (3.1-7.9) | |  |  |
| Female sex, n (%) | 16 (44%) | |  |  |
|  | **Study eyes** (*n* = 36) | | | |
| Best-corrected visual acuity, logMAR | -0.03 ± 0.13 (-0.30 to 0.10) | -0.05 ± 0.11 (-0.30 to 0.10) | -0.02 ± 0.16 (-0.3 to 0.24) | 0.508^‡^ |
| Intraocular pressure, mmHg | 11.2 ± 2.0 (8.0-16.0) | 11.2 ± 1.4 (8.5-14.0) | 0.02 ± 1.5 (-3.0 to 2.5) | 0.910^†^ |
| Spherical equivalent, diopters | -1.3 ± 0.9 (-3.5 to 0.0) | -4.3 ± 0.8 (-6.1 to -3.0) | -2.98 ± 0.8 (-4.5 to -2.0) | < 0.001^†^ |
| Axial length^*^, mm | 23.8 ± 0.8 (23.4 to 24.0) | 25.2 ± 0.6 (24.1 to 26.5) | - | - |
| Cup-to-disc ratio measured in fundus photography | 0.5 ± 0.1 (0.2-0.7) | 0.5 ± 0.1 (0.2-0.7) | 0.01 ± 0.06 (-0.1 to 0.1) | 0.474^‡^ |
| Cup-to-disc ratio measured by cirrus HD-OCT | 0.52 ± 0.12 (0.22-0.71) | 0.53 ± 0.12 (0.22-0.73) | 0.01 ± 0.005 (-0.1 to 0.1) | 0.547^‡^ |
| Tilt ratio measured in fundus photography | 1.07 ± 0.06 (1.00-1.20) | 1.21 ± 0.07 (1.05-1.35) | 0.13 ± 0.06 (0.05-0.30) | < 0.001^†^ |
| Tilt ratio based on OCT-detected disc margin | 1.09 ± 0.07 (1.00-1.23) | 1.21 ± 0.08 00(1.07-1.41) | 0.12 ± 0.08 (-0.04 to 0.31) | < 0.001^†^ |
| Torsion angle, degree in absolute value | 2.21 ± 1.26 (0.20-5.80) | 8.86 ± 2.86 (2.00-14.60) | 6.65 ± 2.74 (0.75-12.96) | < 0.001^†^ |
| LogMAR, logarithm of the minimum angle of resolution; HD-OCT, high-definition optical coherence tomography.  Values are mean ± standard deviation (range).  ^*^ Measurements from 14 eyes in the baseline and 27 in the last visit.  ^†^ Paired t-test, ^‡^ Wilcoxon signed rank test. | | | | |

| **eTable 2.** Circumpapillary Retinal Nerve Fiber Layer Thickness Measures at Study Entry and Completion | | | | |
| --- | --- | --- | --- | --- |
|  | **Study eyes** (*n* = 36) | | | |
|  | Baseline  (*First visit*) | Follow up  (*Last visit*) | Change *(Last visit - First visit)* | *P* value^*^ |
| Global (µm) | 97.7 ± 3.26 (93-104) | 96.8 ± 2.59 (92-104) | -0.78 ± 1.84 (-4 to 4) | 0.007 |
| Quadrants (µm) |  | | | |
| *Superior* | 121.6 ± 6.28 (108-131) | 112.8 ± 7.04 (100-123) | -8.8 ± 7.89 (-31 to 0) | < 0.001 |
| *Temporal* | 75.4 ± 7.65 (62-98) | 94.7 ± 9.44 (80-118) | 19.3 ± 6.01 (13 to 20) | < 0.001 |
| *Inferior* | 128.04± 5.72 (113-139) | 122.1 ± 8.48 (104-139) | -6.31 ± 8.56 (-20 to 13) | < 0.001 |
| *Nasal* | 65.2 ± 8.61 (45-79) | 57.5 ± 8.38 (40-73) | -7.67 ± 7.93 (-28 to 4) | < 0.001 |
| Clock hour (µm) |  | | | |
| *12* | 103.3 ± 8.1 (89-119) | 103.3 ± 9.5 (65-114) | 0.1 ± 12.3 (-32 to 25) | 0.879 |
| *1* | 121.7 ± 12.1 (91-145) | 85.3 ± 13.6 (52-109) | -35.8 ± 17.2 (-72 to 3) | < 0.001 |
| *2* | 74.2 ± 12.9 (51-93) | 65.2 ± 9.68 (45-85) | -8.9 ± 12.8 (-36 to 17) | < 0.001 |
| *3* | 54.1 ± 7.3 (37-68) | 50.4 ± 8.02 (35-66) | -4.3 ± 7.3 (-22 to 9) | 0.011 |
| *4* | 67.2 ± 11.9 (44-96) | 57.1 ± 10.0 (40-78) | -10.2 ± 11.9 (-38 to 11) | < 0.001 |
| *5* | 111.6 ± 5.0 (98-121) | 72.5 ± 5.3 (62-85) | -38.7 ± 5.8 (-49 to -28) | < 0.001 |
| *6* | 127.5 ± 7.5 (106-138) | 126.6 ± 9.7 (110-145) | -1.4 ± 10.1 (-18 to 19) | 0.504 |
| *7* | 146.2 ± 9.0 (128-163) | 167.8 ± 13.5 (140-196) | 22.4 ± 13.9 (1 to 52) | < 0.001 |
| *8* | 81.1 ± 9.7 (67-112) | 99.1 ± 9.7 (84-127) | 18.0 ± 7.9 (1 to 40) | < 0.001 |
| *9* | 53.9 ± 6.6 (41-67) | 73.9 ± 8.8 (62-102) | 20.4 ± 8.6 (9 to 44) | < 0.001 |
| *10* | 91.1 ± 11.7 (70-124) | 111.4 ± 15.9 (88-139) | 19.7 ± 9.0 (-2 to 38) | < 0.001 |
| *11* | 141.1 ± 12.4 (123-171) | 150.1 ± 9.7 (122-170) | 9.7 ± 11.2 (-11 to 29) | < 0.001 |
| Values are mean ± standard deviation (range). ^*^ Paired t-test. *P* < 0.002 (Bonferroni correction for multiple comparisons) was regarded as statistically significant. | | | | |

| **eTable 3.** Proportion of Quadrant Circumpapillary Retinal Nerve Fiber Layer Thickness Distribution at Study Entry and Completion | | | | |
| --- | --- | --- | --- | --- |
|  | **Study eyes** (*n* = 36) | | | |
|  | Baseline  (*First visit*) | Follow up  (*Last visit*) | Change *(Last visit - First visit)* | *P* value* |
| Quadrants (%) |  | | | |
| *Superior* | 31.2 ± 1.29 (27.8-33.5) | 29.3 ± 1.77 (24.8-31.7) | -1.99 ± 1.85 (-7.42 to 0.22) | < 0.001 |
| *Temporal* | 19.2 ± 1.86 (16.5-23.8) | 24.4 ± 2.30 (20.4-30.2) | 5.17 ± 1.10 (3.52 to 9.35) | < 0.001 |
| *Inferior* | 32.8 ± 1.16 (30.0-36.1) | 31.6 ± 2.00 (27.4-35.5) | -1.28 ± 2.02 (-4.76 to 3.23) | 0.001 |
| *Nasal* | 16.7 ± 2.04 (11.7-20.8) | 14.8 ± 2.07 (10.8-19.5) | -1.90 ± 2.12 (-7.19 to 1.12) | < 0.001 |
| Values are mean ± standard deviation (range). ^*^ Paired t-test. *P* < 0.01 (Bonferroni correction for multiple comparisons) was regarded as statistically significant. | | | | |

| **eTable 4.** Inter-examiner Reproducibility of SD-OCT Scan-circle Repositioning | | | | |  |
| --- | --- | --- | --- | --- | --- |
|  | Exam 1 | Exam 2 | ICC | 95% CIs | *P* |
| cpRNFLT (µm) | | | | |  |
| *Average* | 96.9 ± 3.10 | 97.2 ± 3.74 | 0.839 | 0.670-0.921 | < 0.001 |
| *Superior* | 120.6 ± 5.35 | 119.5 ± 4.88 | 0.876 | 0.747-0.940 | < 0.001 |
| *Temporal* | 75.4 ± 7.45 | 79.8 ± 2.62 | 0.767 | 0.641-0.872 | < 0.001 |
| *Inferior* | 127.2 ± 5.73 | 124.8 ± 6.11 | 0.799 | 0.589-0.902 | < 0.001 |
| *Nasal* | 64.3 ± 8.24 | 64.7 ± 6.27 | 0.848 | 0.689-0.926 | < 0.001 |
| cpRNFLT proportion (%) | | | | |  |
| *Superior* | 31.1 ± 1.24 | 30.8 ± 1.57 | 0.784 | 0.684-0.877 | < 0.001 |
| *Temporal* | 19.5 ± 1.80 | 20.5 ± 2.66 | 0.747 | 0.581-0.876 | < 0.001 |
| *Inferior* | 32.8 ± 1.15 | 32.1 ± 1.58 | 0.776 | 0.641-0.891 | < 0.001 |
| *Nasal* | 16.6 ± 1.98 | 16.6 ± 1.47 | 0.863 | 0.719-0.833 | < 0.001 |
| Values are mean ± standard deviation. ICC; intra-class correlation coefficient, CIs; confidence intervals, cpRNFLT; circumpapillary retinal nerve fiber layer thickness. | | | | | |

| **eTable 5.** Intra-examiner Reproducibility of SD-OCT Scan-circle Repositioning | | | | |  |
| --- | --- | --- | --- | --- | --- |
|  | Examiner 1 | Examiner 2 | ICC | 95% CIs | *P* |
| cpRNFLT (µm) | | | | |  |
| *Average* | 96.9 ± 3.10 | 96.7 ± 3.36 | 0.956 | 0.909-0.978 | < 0.001 |
| *Superior* | 120.6 ± 5.35 | 120.3 ± 5.61 | 0.955 | 0.908-0.978 | < 0.001 |
| *Temporal* | 75.4 ± 7.45 | 75.2 ± 7.44 | 0.983 | 0.965-0.992 | < 0.001 |
| *Inferior* | 127.2 ± 5.73 | 126.2 ± 6.43 | 0.951 | 0.899-0.976 | < 0.001 |
| *Nasal* | 64.3 ± 8.24 | 65.0 ± 8.33 | 0.919 | 0.834-0.961 | < 0.001 |
| cpRNFLT proportion (%) | | | | |  |
| *Superior* | 31.1 ± 1.24 | 31.1 ± 1.26 | 0.943 | 0.883-0.972 | < 0.001 |
| *Temporal* | 19.5 ± 1.80 | 19.4 ± 1.81 | 0.980 | 0.959-0.990 | < 0.001 |
| *Inferior* | 32.8 ± 1.15 | 32.6 ± 1.29 | 0.898 | 0.792-0.950 | < 0.001 |
| *Nasal* | 16.6 ± 1.98 | 16.8 ± 1.99 | 0.927 | 0.851-0.964 | < 0.001 |
| Values are mean ± standard deviation. ICC; intra-class correlation coefficient, CIs; confidence intervals, cpRNFLT; circumpapillary retinal nerve fiber layer thickness. | | | | | |
